# Non-suicidal self-injury and professional psychological help-seeking among Chinese left-behind children: prevalence and influencing factors

**DOI:** 10.1186/s12888-023-04801-0

**Published:** 2023-04-26

**Authors:** Na Yong, Jing Luo, Jia-ming Luo, Yi-song Yao, Jing Wu, Han Yang, Jing-dan Li, Shuang Yang, Yuan-yuan Leng, Hong-chuan Zheng, Yang Fan, Ying-dan Hu, Jin Ma, Ya-wen Tan, Ji-yang Pan

**Affiliations:** 1 Department of Psychiatry, The First Affiliated Hospital of Jinan University, Guangzhou, Guangdong China; 2 School of Psychiatry, North Sichuan Medical College, Nanchong, Sichuan China; 3 Department of Child and Adolescent Psychology, Nanchong Psychosomatic Hospital, Nanchong, Sichuan China; 4 Mental Health Center, Southwest Petroleum University, Nanchong, Sichuan China

**Keywords:** Non-suicidal self-injury, Left-behind children, Coping styles, Help-seeking, Prevalence, Influencing factors

## Abstract

**Background:**

Non-suicidal self-injury (NSSI) is a risk factor for suicide. This study aimed to investigate the prevalence of NSSI and professional psychological help-seeking status and influencing factors among left-behind children (LBC) in China.

**Methods:**

We implemented a population-based cross-sectional study in participants aged 10–18 years. Sociodemographic characteristics, NSSI, help-seeking status and coping style were measured by self-reported questionnaires. A total of 16,866 valid questionnaires were collected, including 6096 LBC. Binary logistic regression models were used to analyze the factors influencing NSSI and professional psychological help-seeking.

**Results:**

The incidence of NSSI among LBC was 4.6%, significantly higher than that of non-left-behind children (NLBC). This incidence was higher among girls. Moreover, 53.9% of LBC with NSSI did not receive any treatment and only 22.0% sought professional psychological help. LBC often adopt emotion-oriented coping styles, specifically, those with NSSI. LBC with NSSI who seek professional help tend to adopt problem-oriented coping styles. Logistic regression analysis revealed that girls, learning stage, single-parent, remarried families, patience, and emotional venting were risk factors for NSSI in LBC, while problem-solving and social support seeking were protective factors. Moreover, problem-solving was also a predictor for seeking professional psychological help, patience will prevent it.

**Limitations:**

This was an online survey.

**Conclusions:**

The prevalence of NSSI in LBC is high. Gender, grade, family structure, and coping style affect the occurrence of NSSI among LBC. Only a few LBC with NSSI seek professional psychological help, while the coping style will affect the help-seeking behavior.

## Introduction

Left-behind children (LBC) are rural children younger than 18 years who are left at home when one or both parents migrate to the city for work [1]. In 2013, there were nearly 68 million LBC in China, accounting for 21.9% of the child population [2]. Due to the rapid urbanization in China, numerous rural laborers migrated to cities for higher-paying jobs and some chose to leave their children in their hometown [3]. Long-term separation from their parents, lack of family love, and ineffective supervision can put LBC at risk of mental health problems and even extreme behaviors [4, 5]. In particular, LBC may face higher risk of depression and self-harm than non-left-behind children (NLBC) [6, 7]. Among them, non-suicidal self-injury (NSSI) is a serious behavior problem attracting the attention of researchers [8].

NSSI refers to the behavior of directly, intentionally, and repeatedly injuring one's body without the intent to die; the common forms include beating, cutting, burning, hitting, and scratching [9]. NSSI is a common but perplexing phenomenon worldwide. It is highly prevalent among adolescents and studies have found that the lifetime prevalence of NSSI ranges from 17%–60% [10]. A systematic review showed that its prevalence rates peak between 15 to 17 years and decline from young to middle adulthood [11]. However, its prevalence among LBC is much higher than that of NLBC [12]. LBC face long-term parent–child separation during their school years, and the absence makes them more likely to have problematic behaviors, resulting in unfortunate consequences. Research shows that family environment is important for teenagers' mental health and family dysfunction can effectively predict NSSI’s occurrence [13]. Although it is essentially different from suicidal behavior, it is a risk factor for suicide attempts and also the strongest predictor of completed suicide [14, 15]. In China, suicide is the leading cause of death among young people aged 15–34 years [16]. Moreover, NSSI is also related to adverse outcomes in adulthood, such as increasing the risk of mental illness and substance abuse [17]. The function of self-injury is to avoid or escape the internal experience(s) that individuals do not want or hate, which mainly refers to avoiding negative emotional experience(s) [18]. Previous research identified that depressive symptoms, stressors, and poor relationship quality are risk factors for NSSI [19].

When a psychological crisis occurs, asking for help is an effective coping strategy, which can promote mental health. Help-seeking refers to the behavior of actively seeking help to gain understanding, advice, treatment, and general support during distressing experiences. This includes informal help-seeking, which refers to seeking help from friends and family and formal help-seeking, which is seeking help from professional sources, such as mental health and health professionals, who have professional training in providing help and advice [20]. Research has indicated that the latter are effective in relieving an individual’s psychological distress [21]. Unfortunately, only a few children and adolescents seek professional help for mental health problems [22]. A study showed that less than 40% of adolescents with depressive symptoms receive professional treatment in the US [23]. Furthermore, compared with adults, children and adolescents are less likely to seek medical help. Untreated depression can have a lasting impact on their personal, social, and academic performance [24]. However, not asking for help even when suffering from serious psychiatric problems is an evasive coping strategy, which will negatively impact the learning state, interpersonal relationships, and life. Furthermore, untreated mental health issues hinder personality development and the improvement of mental health. There are many LBC in China and research shows that the incidence of NSSI is higher, specifically, in LBC [12], but it is unclear whether they seek professional psychological help in time.

Coping refers to a person attempting to regulate specific stressful situations as well as thoughts, emotions, physiology, and behaviors during stressful events or circumstances [25]. Coping styles include positive (PC) and negative coping style (NC) components. PC means seeking help to solve problems or optimistically facing stressful situations, while NC refers to coping with stress through fantasy, denial, or unhealthy behavior [26]. PC are associated with better health [27], whereas NC with poor health outcomes and social dysfunction [28]. The coping style formed during adolescence determines the same in adulthood [29]. Studies have shown that adolescents who use PC such as problem-solving can better deal with the challenges than those who use NC such as avoidance [30]. NSSI is essentially a coping strategy with maladjustment [31]. It can be explained as a way to regulate tension. Research shows that those who often adopt NC are prone to NSSI when dealing with negative emotions [32]. Teenagers may cope with the increasing psychological distress through NSSI, which shows different psychological symptoms [33], such as anxiety, depression, and behavioral problems [34, 35]*.* Especially for girls, studies reported that girls are more likely to engage in NSSI than boys [36], as female adolescents seem to use NC more than males [37]. However, it is unclear whether coping styles affect professional help-seeking behavior, specifically, among LBC with NSSI.

There are many studies on the NSSI behavior of LBC. However, due to regional, ethnic, and survey tool(s) differences, the survey results are significantly different, with prevalence rates ranging from 3.1% to as high as 60% [8, 38], and there are less data in China and most are concentrated in specific communities such as ethnic minorities[7]. Furthermore, NSSI behavior is harmful to individuals. At present, since there is no study on professional psychological help-seeking for LBC with NSSI, we first investigated its status and influencing factors to provide a basis for improving the mental health of LBC in China.

## Methods

### Participants and procedures

The survey report shows that the distribution of LBC is significantly uneven in China. It is mainly concentrated in Sichuan, Henan, Anhui, and other major labor export provinces, specifically, in Sichuan and Henan where its proportion has reached 11.3% and 10.7%, respectively [39]. Our study was implemented in Sichuan, which is a southwest province of mainland China. Yingshan, located in the northeastern area of Sichuan Basin and it is a key area for rural LBC in Sichuan, where is poverty-stricken and its provinces are densely populated by inhabitants of the Han Nationality with 970,000 people across 26 townships, including 96 schools of different levels.

We selected Yingshan as our research region. From May to September 2020, using the random cluster sampling method, we extracted data from all primary and secondary schools (junior and senior secondary) in eight towns as the research object. Since some rural students moved to the county town for better education, we also extracted data from three primary and three secondary schools in the country town. The survey was conducted using online questionnaires after receiving informed consent from the students and their parents. A total of 19,005 questionnaires were collected from participants. After excluding incomplete questionnaires, there were 16,968 valid questionnaires, and the valid response rate was 89.3% (16,968/19,005). The research proposal was approved by the ethics committee of the Physical and Mental Hospital of Nanchong City (The Sixth People’s Hospital of Nanchong City).

Inclusion criteria for LBC were: 1) Rural children younger than 18 years who were left at home after one or both migrated parents; 2) Separation exceeded six consecutive months in the last year; 3) Students from grade 4 of primary school to grade 3 of senior high school.

Exclusion criteria were: As the literature suggested that until the age of 10, one does not understand the concepts of death and suicide [40], we excluded children under 10 years. Moreover, we excluded those who were unwilling to participate in the survey.

### Measures

#### Sociodemographic characteristics

A self-developed structured questionnaire was used to collect sociodemographic characteristics, including gender (boys/girls), age, grade, only child (yes/no), LBC (yes/no), residence (rurality/ urban), boarder (yes/no), parents’ education levels (less than junior middle school, junior middle school, senior middle school, college or more), family structure type (nuclear /single parent /remarried).

#### *Ottawa Self-Injury Inventory (OSI)* [41]

A self-report questionnaire used to assess the occurrence, frequency, motivation, types, functions, addictive features, severity indicators, and type of help-seeking of LBC with NSSI. The Chinese version of the Ottawa self-injury scale has good test–retest reliability and structural validity, and the test–retest reliability coefficient was above 0.400. The internal consistency of Cronbach's α coefficient of function subscales was 0.952 and that of its four factors ranged from 0.637–0.896 [42]. It is a comprehensive evaluation tool for NSSI behavior. This study investigated the NSSI that occurred in the past or present without suicidal purpose. A behavior was considered as self-injurious if the self-harm occurred once or more. Meanwhile, the items on help-seeking were used in the questionnaire to evaluate the status regarding LBC with NSSI (e.g., What treatments have you received for self-injurious behavior? 1) I have not received any treatment, 2) I refused treatment, 3) Self-help, 4) Individual treatment, 5) Psychology consult at school, 6) Group psychotherapy, 7) Family psychotherapy, 8) Medication).

#### Coping Style Scale for Middle School Students(CSSMSS) [43]

It is compiled by China and used to measure the coping style among adolescents. It comprises 36 items and is divided into seven factors, including problem-solving, social support seeking, positive and reasonable explanation, patience, escape, venting, and fantasy denial. Among them, the first three coping styles are problem-oriented, and the last four are emotion-oriented. Problem-oriented coping refers to dealing with the problems that cause trouble and changes the relationship between individual and environment; emotional-coping aims to directly adjust emotions and reduce the emotional pain. In many studies, it is summarized as PC and NC. All the items were rated on a four-point scale. It has good test–retest reliability and structural validity, as the test–retest reliability coefficient was 0.89 and Cronbach’s α for the subscale was 0.92.

### Statistical analysis

All analyses were conducted by SPSS (version 20.0) software. Descriptive statistics were employed to delineate and compare general characteristics. Chi-square test was used for categorical variables, and the t-test was used to assess the differences between LBC and NLBC. We used binomial logistic regression models to test the associations of NSSI with PC and NC and professional psychological help-seeking behavior. When analyzing the influencing factors for NSSI and professional psychological help-seeking among LBC with NSSI, we used binary logistic regression with a backward progression. The significance level was set at a two-tailed probability of *P* < 0.05.

## Results

### Demographic characteristics

Among the total sample of 16,866 participants, LBC accounted for 36.1% (6,096/16,866), with 52.8% boys (3,221/ 6,096) and 47.2% (2,875/6,096) girls. Participants’ age ranged from 10–18 years, with an average age of (13.51 ± 2.32) years. Primary school students accounted for 41.5% (2,532/6,096), junior middle school students for 35.5% (2,164/6,096), and senior middle school students for 23.0% (1,400/6,096). In China, since primary and junior middle education is compulsory, the number of students in these two stages is significantly more than those in senior middle school. However, we observed significant differences in many demographic characteristics between LBC and NLBC. For example, the proportion of LBC in senior high school was significantly higher than NLBC (*P* < 0.001); most LBC live in rural areas (58.7%), and there were significantly more LBC than NLBC in single-parent and remarried families than nuclear families (see Table 1).

**Table 1:** The demographic characteristics of the LBC and NLBC groups

Variables	Total (*n* = 16,866)	NLBC (*n* = 10,770)	LBC (*n* = 6,096)	*χ2*	*p*
**Gender** **: ** **N (%)**					
Boy	8,850(52.5)	5,629(52.3)	3,221(52.8)	0.512	0.480
Girl	8,016(47.5)	5,141(47.7)	2,875(47.2)		
**Learning stage** **: ** **N (%)**					
Primary school	8,222(48.7)	5,690(52.8)	2,532(41.5)	460.163	0.000
Junior middle school	6,051(35.9)	3,887(36.1)	2,164(35.5)		
Senior middle school	2,593(15.4)	1,193(11.1)	1,400(23.0)		
**Residence** **: ** **N (%)**					
County town	9,893(58.7)	7,374(68.5)	2,519(41.3)	1.181	0.000
Rural area	6,973(41.3)	3,396(31.5)	3,577(58.7)		
**Boarder** **: ** **N (%)**					
No	14,047(83.3)	9,702(90.1)	4,345(71.3)	9.894	0.000
Yes	2,819(16.7)	1,068(9.9)	1,751(28.7)		
**Only child** **: ** **N (%)**					
No	14,498(86.0)	9,380(87.1)	5,118(84.0)	31.741	0.000
Yes	2,368(14.0)	1,390(12.9)	978(16.0)		
**Family structure types** **: ** **N (%)**					
Nuclear families	14,701(87.2)	9,861(91.6)	4,840(79.4)	531.532	0.000
Single-parent families	1,342(8.0)	519(4.8)	823(13.5)		
Remarried families	823(4.8)	390(3.6)	433(7.1)		

*NLBC* Non-left behind children, *LBC* Left-behind children

### Detection of NSSI among LBC

Our study found that the incidence rate of NSSI among LBC was 4.6% (282/6,096), and this tendency was higher in girls than boys, with an OR 2.14 (95% CI: 1.67–2.75). Moreover, significant differences were found between LBC and NLBC groups in boys and girls regarding the prevalence of NSSI (*P* < 0.05). Along with the increase of learning stage, the risk of incident NSSI significantly increased; the detection rate in LBC was significantly higher than in NLBC only in junior middle school (5.6% versus, 4.4%, *P* < 0.05). The detection rate of NSSI in LBC was significantly higher than that of NLBC living in county towns (3.1% versus, 4.5%, *P* < 0.001). The incidence rate among boarder students was significantly higher than non-boarder students regarding LBC, with OR 1.71 (95% CI:1.33–2.18). The incidence rate was significantly increased in single-parent and remarried families among LBC; however, the detection rate of NSSI only in nuclear families was significantly higher in LBC than that of NLBC (*P* < 0.05)(see Table 2).

**Table 2:** Detection of NSSI among LBC and NLBC

Variables	LBC (*n* = 6,096)	OR(95%CI)	NLBC(*n* = 10,770)	*χ2*	*p*
**Total (N, %)**	282(4.6)		359(3.3)	17.791	0.000
**Gender(N, %)**					
Boy	99(3.1)	1	126(2.2)	5.767	0.016
Girl	183(6.4)	2.14 (1.67–2.75)**	233(4.5)	12.591	0.000
**Learning stage (N, %)**					
Primary school	55(2.2)	1	100(1.8)	1.629	0.202
Junior middle school	122(5.6)	2.69 (1.95–3.72)**	172(4.4)	4.422	0.035
Senior middle school	105(7.5)	3.65 (2.62–5.10)**	87(7.3)	0.040	0.841
**Residence (N, %)**					
County town	114(4.5)	1	228(3.1)	11.563	0.001
Rural area	168(4.7)	1.04 (0.82–1.33)	131(3.9)	2.989	0.084
**Boarder (N, %)**					
No	169(3.9)	1	279(2.9)	9.990	0.002
Yes	113(6.5)	1.71 (1.33–2.19)**	80(7.5)	1.119	0.290
**Only child (N, %)**					
No	231(4.5)	1	316(3.4)	11.949	0.001
Yes	51(5.2)	0.34 (0.85–1.59)	43(3.1)	6.776	0.009
**Family structure types (N, %)**					
Nuclear families	184(3.8)	1	298(3.0)	6.223	0.013
Single-parent families	62(7.5)	2.06 (1.53–2.78)**	26(5.0)	3.309	0.068
Remarried families	36(8.3)	2.30 (1.58–3.34)**	25(6.4)	1.084	0.298

*NSSI* Non-suicidal self-injury, *NLBC* Non-left behind children, *LBC* Left-behind children

*P*: Difference significance test of the prevalence rate of NSSI between subgroups in the LBC and NLBC group

^**^ *P* < 0.01

### Help-seeking status of LBC with NSSI

Among LBC with NSSI, 53.9% did not receive any treatment, 9.2% refused treatment, 14.9% used self-help, and only 22.0% sought professional psychological assistance. There was no significant difference regarding help-seeking status between LBC and NLBC with NSSI (*P* > 0.05), (see Table 3).

**Table 3:** Help-seeking status of LBC and NLBC with NSSI

Variables	LBC (*n* = 282)	NLBC (*n* = 359)	*χ*^*2*^	*p*
Don't receive any treatment	152(53.9)	182(50.7)	0.650	0.420
Refuse treatment	26(9.2)	43(12.0)	1.251	0.263
Self-help	42(14.9)	65(18.1)	1.172	0.279
Professional psychological help	62(22.0)	69(19.2)	0.743	0.389

*NSSI* Non-suicidal self-injury, *NLBC* Non-left behind children, LBC Left-behind children

There was no significant difference among different demographic characteristics regarding professional psychological help-seeking in LBC, subgroups in the LBC and NLBC with NSSI (see Table 4).

**Table 4:** Professional psychological help-seeking status among LBC with NSSI and compared with NLBC with NSSI

	LBC with NSSI		NLBC with NSSI	*χ*^*2*^	*p*
Variables	Help-seeking (%)	OR(95%CI)	Help-seeking (%)		
**Gender **(N, %)					
Boy	22(22.2)	1	28(22.2)	0.000	1.000
Girl	40(21.9)	0.98 (0.54–1.77)	41(17.6)	1.187	0.276
**Learning stage** (N, %)					
Primary school	14(25.5)	1	20(21.9)	0.617	0.432
Junior middle school	24(19.7)	0.72 (0.34–1.52)	32(18.6)	0.053	0.818
Senior middle school	24(22.9)	0.81 (0.41–1.85)	17(19.5)	0.312	0.577
**Residence**					
County town	24(21.1)	1	39(17.1)	0.788	0.375
Rural area	38(22.6)	1.10 (0.62–1.95)	30(22.9)	0.003	0.954
**Boarder** (N, %)					
No	36(21.3)		52(18.6)	0.473	0.492
Yes	26(23.0)	1.10 (0.62–1.96)	17(21.2)	0.084	0.772
**Only child** (N, %)					
No	49(21.2)	1	60(19.0)	0.414	0.520
Yes	13(25.5)	1.27 (0.63–2.57)	9(20.9)	0.271	0.603
**Family structure types** (N, %)					
Nuclear families	38(20.7)	1	60(20.1)	0.019	0.891
Single-parent families	14(22.6)	1.12 (0.56–2.24)	7(26.9)	0.190	0.663
Remarried families	10(27.8)	1.48 (0.66–3.33)	3(12.0)	2.190	0.139

*NSSI* Non-suicidal self-injury, *NLBC* Non-left behind children, *LBC* Left-behind children

*P*: Difference significance test of the professional psychological help-seeking status between subgroups in the LBC and NLBC with NSSI

### Coping styles of LBC

The results showed that the scores of problem-solving, positive rational explanation, and the total score of problem-oriented coping among LBC were significantly lower than those of NLBC (*P* < 0.001), while the scores of patience, evasion, emotional venting, fantasy/denial, and the total score of emotion-oriented coping were significantly higher than those of NLBC (*P* < 0.01). Only evasion differed significantly by gender among LBC, scoring significantly higher in boys (*P* < 0.05) (see Table 5).

**Table 5:** Comparison of coping styles

	LBC (*n* = 6,096)	NLBC (*n* = 10,770)	*t*	*P*	LBC		*t*	*P*
					Boy (*n* = 3,221)	Girl (*n* = 2,875)		
Problem-solving	19.42 ± 5.63	19.82 ± 5.70	-4.497	0.000	19.52 ± 5.71	19.30 ± 5.53	1.484	0.138
Social support seeking	17.31 ± 5.05	17.40 ± 5.12	-1.026	0.305	17.32 ± 5.13	17.31 ± 4.94	0.092	0.927
Positive rational explanation	13.23 ± 3.97	13.50 ± 4.03	-4.253	0.000	13.27 ± 4.00	13.19 ± 3.92	0.698	0.485
Problem-oriented coping(total score)	49.96 ± 13.60	50.72 ± 13.74	-3.490	0.000	50.10 ± 13.87	49.81 ± 13.29	0.852	0.394
Patience	8.75 ± 2.90	8.57 ± 2.89	3.916	0.000	8.79 ± 2.91	8.71 ± 2.87	1.124	0.261
Evasion	7.35 ± 2.82	7.21 ± 2.75	3.143	0.002	7.42 ± 2.88	7.27 ± 2.75	2.003	0.045
Emotional venting	7.36 ± 2.91	7.12 ± 2.87	5.234	0.000	7.39 ± 2.92	7.33 ± 2.90	0.687	0.492
Fantasy/Denial	9.25 ± 3.67	8.95 ± 3.56	5.300	0.000	9.28 ± 3.72	9.22 ± 3.62	0.626	0.532
Emotion-oriented coping (total score)	32.72 ± 10.49	31.85 ± 10.19	5.265	0.000	32.88 ± 10.72	32.54 ± 10.22	1.258	0.209

*NLBC* Non-left behind children, *LBC* Left-behind children

### Differences of coping styles in LBC with NSSI and whether to seek professional help

The scores of problem-solving, social support seeking, positive rational explanation, and the total score of problem-oriented coping were significantly lower in LBC with NSSI (*P* < 0.05), whereas the scores of patience, evasion, emotional venting, fantasy/denial, and the total score of emotion-oriented coping were significantly higher in LBC with NSSI (*P* < 0.05) (see Table 6).

**Table 6:** Differences of coping styles in LBC with NSSI and whether they sought professional help

	LBC with NSSI		*t*	*P*	Professional psychological help-seeking in LBC with NSSI		*t*	*P*
	Yes	No			Yes	No		
Problem-solving	17.22 ± 5.06	19.52 ± 5.63	-6.743	0.000	18.46 ± 4.64	16.98 ± 4.61	2.479	0.014
Social support seeking	16.21 ± 4.68	17.37 ± 5.06	-3.760	0.000	17.47 ± 4.73	15.40 ± 4.65	3.441	0.001
Positive rational explanation	12.25 ± 3.68	13.28 ± 3.97	-4.271	0.000	13.20 ± 3.51	12.06 ± 3.42	2.560	0.011
Problem-oriented coping(total score)	45.68 ± 12.17	50.17 ± 13.63	-5.432	0.000	49.13 ± 11.95	44.44 ± 11.01	3.236	0.001
Patience	10.13 ± 2.83	8.69 ± 2.88	8.234	0.000	10.04 ± 3.01	9.86 ± 2.67	-0.307	0.416
Evasion	8.43 ± 2.70	7.30 ± 2.81	6.606	0.000	8.37 ± 2.69	8.44 ± 2.71	-0.848	0.074
Emotional venting	8.90 ± 3.25	7.29 ± 2.87	9.150	0.000	8.47 ± 3.24	9.02 ± 3.25	-1.188	0.236
Fantasy/Denial	10.90 ± 3.66	9.17 ± 3.65	7.763	0.000	10.42 ± 3.56	11.04 ± 3.69	-1.172	0.242
Emotion-oriented coping(total score)	38.36 ± 9.92	32.44 ± 10.44	9.320	0.000	37.07 ± 10.03	38.73 ± 9.89	-1.166	0.245

*NSSI* Non-suicidal self-injury, LBC Left-behind children

The total score of problem-oriented coping and score of each factor were significantly higher in LBC with NSSI who sought professional help (*P* < 0.05) (see Table 6).

### Risk factors for NSSI in LBC

Logistic regression analysis revealed that girls, learning stage, and single-parent and remarried families were risk factors for NSSI. The coping styles of patience and emotional venting were also risk factors, whereas the coping styles of problem-solving and social support seeking were protective factors. (shown in Table 7).

**Table 7:** Risk factors for NSSI in LBC, analyzed by binary backward logistic regression

Variance	β	S.E	Wald*x*^*2*^	*P*	*OR*(95% *CI)*
**Gender**					
Boy	1				
Girl	0.808	0.132	37.418	0.000	2.24(1.73–2.91)
**Learning stage(N, %)**					
Primary school	1				
Junior middle school	0.728	0.169	18.454	0.000	2.07(1.49–2.89)
Senior middle school	0.959	0.176	29.673	0.000	2.61(1.85–3.68)
**Family structure types**					
Nuclear families	1				
Single-parent families	0.559	0.159	12.416	0.000	1.75(1.28–2.39)
Remarried families	0.505	0.201	6.309	0.012	1.66(1.12–2.46)
**Problem-solving**	-0.097	0.020	23.565	0.000	0.91(0.87–0.94)
**Social support seeking**	-0.060	0.022	7.434	0.006	0.94(0.90–0.98)
**Patience**	0.211	0.029	54.269	0.000	1.24(1.17–1.31)
**Emotional venting**	0.122	0.025	23.310	0.000	1.13(1.08–1.19)

*SE* Standard Error, *OR* Odds ratio, *CI* Confidence Interval, *NSSI* Non-suicidal self-injury, *LBC* Left-behind children.

### Predictors for professional psychological help-seeking in LBC with NSSI

Logistic regression analysis revealed that the coping style of problem-solving was a predictor of professional psychological help-seeking, while that of patience hindered psychological help-seeking among LBC with NSSI (see Table 8).

**Table 8:** Predictors for professional psychological help-seeking in LBC with NSSI, analyzed by binary backward logistic regression

Variance	β	S.E	Wald*x*^*2*^	*P*	*OR*(95% *CI)*
Problem-solving	0.096	0.033	8.679	0.003	1.10 (1.03 ~ 1.17)
Patience	-0.130	0.059	4.858	0.028	0.89 (0.78 ~ 0.99)

*SE* Standard Error, *OR* Odds ratio, *CI* Confidence Interval, *NSSI* Non-suicidal self-injury, *LBC* Left-behind children

## Discussion

### Incidence of NSSI in LBC

Self-injurious behavior has become a social problem that can have serious effects on teenagers’ health [44, 45]. Long-term and repeated self-injurious behavior causes direct trauma to the body, is not conducive to teenagers’ physical and mental health, and can cause a heavy burden to the family and social medical undertakings.

This study found that the incidence of NSSI of LBC is 4.6%, which is significantly higher than that of NLBC in the region, but lower than the survey results of LBC in Guizhou Province (33.7%) [46] and Yunnan Province (48%) [7], which have a majority of ethnic minorities in China, and also lower than that in Italy (42%) [14], Britain (18.8%) [47] and Germany (25.6%) [48]. The reasons for the differences could be: 1) Age difference: the participants of our study were 10–18 years old, while that of most other studies were junior or senior high school students. Studies have shown that NSSI behavior increases after the age of 14 years [48]. Our study also supports this conclusion. Our results show that the incidence of NSSI is lowest among primary school students, and along with the increase of school stage, the incidence of NSSI also increases significantly; 2) Racial differences: One study found that the incidence of self-injurious behavior was affected by race and reports show that it is lowest among men in Asia, and highest among black women [49]. However, in China, different ethnic groups have different cultures and religious beliefs, and research indicates that some minority ethnicities have a high suicide rate, which may be related to the local cultural background, imitation learning, folk customs, and religions, such as the viewpoint that the body and soul of humans can be separated. Specifically, while facing difficulties, one may follow suit, because others provide a model of how to deal with setbacks [50]. This study was focused in Sichuan, in inland China where the participants are mainly of Han nationality, in a poor county with simple folk customs, no special religious beliefs, and less exposure to negative information from the outside world. Meanwhile, as there were fewer people with NSSI, it may reduce the imitation and self-injury rates.

This study also found that the detection rate of NSSI in LBC was higher among girls, which is consistent with previous studies [46]. There are also significant differences regarding the incidence rate of NSSI in different family environments; for example, it is significantly higher among single-parent families and reorganized families, which is consistent with previous studies [51]. It is suggested that the family environment may impact the psychological behavior of LBC.

### Psychological help-seeking

Our study found that most LBC who had NSSI did not seek help. For instance, around half did not receive any treatment, 9.2% refused treatment, and only 22% sought professional psychological help. Research shows that when individuals face adverse emotional experiences as a result of negative events, they turn to self-injury due to low emotional management ability, such as low frustration tolerance, poor use of emotional regulation strategies, and unreasonable use of emotional regulation. When they face the same situation, they will choose the same way to avoid negative emotions [52]. Hence, effective emotion regulation can effectively control emotional impulses, alleviate the negative impact of emotions, and maintain the healthy development of mental health [31]. For individuals, the most direct and rapid way to solve emotional problems is to ask for help and attain psychological comfort [21]. Mental health professionals use psychological methods to assist those who have problems in psychological adaptation, and seek to solve them. It is difficult to replace this by using non-professional psychological help [21]. However, the survey shows that rather than seeking professional help, adolescents, and male adolescents, in particular, may attempt to resolve their difficulties alone [53]. Our study also indicates that lower utilization rate of professional psychological resources among LBC with NSSI requires high attention by schools and families.

### Coping styles of LBC

Our study shows that LBC often adopts emotion-oriented coping styles, specifically, those with NSSI, which is part of NC, including patience, evasion, venting emotions, fantasy, and denial. The results are similar to previous studies [54, 55]. Foreign studies pointed out that individuals who often adopt emotion-oriented coping are also more likely to show NSSI behavior when dealing with negative emotions [55]. Another study shows obvious gender differences in adolescents' coping styles; for example, girls prefer to adopt negative coping styles [37], but our study found that only boys scored significantly higher only on evasion. This study indicates avoidance coping style was positively correlated with emotional behavior problems, such as emotional symptoms, conduct problems, and peer relationship problems [56]. Adolescents who use positive coping styles such as problem-solving can better deal with the challenges than those who use negative coping styles such as escape [30].

However, our study found the scores of problem-oriented coping styles, such as problem-solving, social support seeking, and positive rationalization explanation, among LBC with NSSI who sought professional help were significantly higher. Previous studies have shown that the coping strategy type affects individual physical and mental health; negative coping is related to negative effects, whereas positive coping have a positive impact on mental health [57]. Furthermore, our findings show that problem-solving coping strategies seem to be a protective factor for mental health [58]. Our study further suggests that positive coping strategies facilitate professional psychological help-seeking.

### Risk factors for NSSI among LBC

In this study, we found obvious gender differences in the occurrence of NSSI among LBC; the incidence in girls was significantly higher; further, girls had the risk factors for NSSI, which is consistent with previous studies [59]. This is probably due to the difference in physiological mechanisms such as the difference in hormone levels, brain development mechanisms, and other factors between boys and girls. Studies show that self-injurious behavior in adolescents has a correlation with uncomfortable physical reactions regarding menarche and menstruation and girls often use various self-injury methods to deal with them [60]. Specifically, during adolescence, girls experience emotional sensitivity, and strong self-esteem personality traits, resulting in their higher perception of emotions, interpersonal relationships, learning, and other problems [61].

Moreover, learning stage and family structure types were risk factors for NSSI among LBC. Research shows that the integrity of the family structure is the most important for teenagers’ healthy growth among many family-related factors [62]. Children from reorganized families are emotionally unstable and prone to psychological problems such as timidity, inferiority complex, sensitivity, and paranoia; single-parent families show more refusal, denial, punishment, excessive interference, or protection towards their children [63], which can easily affect them psychologically and behaviorally.

Regarding coping styles, patience and emotional venting were also risk factors for NSSI, whereas problem-solving and social support seeking were protective factors. Patience and emotional venting are emotional-coping styles, which are part of the NC style. This is consistent with previous studies, which also suggest that emotional coping is positively correlated with adolescent NSSI [64]. The emotion management function hypothesis also states that when facing pressure, individuals who adopt an emotion-oriented coping style are more likely to alleviate negative emotions through NSSI behavior [18]. Although problem-solving and social support seeking belong to a problem-oriented coping style, there are also inconsistent findings between the relationships between PC and NSSI, for example, studies have not found a significant relationship between them [65]. Our results indicated that we should strengthen psychological services and behavioral intervention for girls, those with poor family structure, and senior students, by increasing PC and decreasing NC to help protect against NSSI.

### Predictors for professional psychological help-seeking in LBC with NSSI

Fewer studies explored the factors for predictors of professional psychological help-seeking, specifically, regarding LBC with NSSI. Through our study, we found that problem-solving was a predictor for professional psychological help-seeking, while patient was a barrier to it. Previous studies have pointed out that PC is associated with better health [27], whereas NC is associated with poor health outcomes and social dysfunction [28]. Therefore, our result supports this theory as problem-solving belongs to PC, and positive professional psychological help-seeking behavior can effectively relieve an individual’s psychological distress [21].

Our study also has several limitations: First, the questionnaire was collected through the Internet, and the age range of our respondents was 10–18 years. As some younger children would be unable to accurately understand all questions, they may have not been able to answer them. Second, since the participants were selected from Sichuan province, the results may be biased; hence, they must be cautiously generalized to all LBC in China. In future research, we can select the multi-centered model, and collect data by issuing questionnaires offline and answering questions on the spot by investigators, which can reduce the data bias.

## Conclusion

Our findings confirmed that the prevalence of NSSI among LBC in mainland China is significantly higher than that of NLBC. The occurrence of NSSI in LBC is related to gender, family structure, the increase of school stage, and coping style. Only a few LBC with NSSI seek professional psychological help. The coping style affects their help-seeking behavior and problem-solving was a predictor for professional psychological help-seeking, while patience will hinder it. NSSI in LBC presents an important public health problem in rural China, and more attention should be paid to prevention and intervention, specifically, those who are unwilling to seek professional psychological assistance. We should encourage LBC to adopt correct and positive coping styles in adversity, specifically, seeking professional psychological assistance can help improve their mental health and prevent the occurrence of NSSI behavior.

## Data Availability

The datasets used and the research protocol that support the findings of this study are available on request from the corresponding author, J. Luo. The data are not publicly available due to their containing information that could compromise the privacy of research participants.

## Abbreviations

LBC: Left-behind children
NLBC: Non-left behind children
NSSI: Non-suicidal self-injury
PC: Positive coping style
NC: Negative coping style
